# Plant Invasions in Mountain Areas: Global and Mediterranean Perspectives

**DOI:** 10.3390/plants15040588

**Published:** 2026-02-13

**Authors:** Neus Nualart, Javier Martínez-Fuentes, Eduard López-Guillén, Jordi López-Pujol

**Affiliations:** 1 Institut Botànic de Barcelona (IBB), CSIC-CMCNB, 08038 Barcelona, Spain; 2 Escuela de Ciencias Ambientales, Universidad Espíritu Santo (UEES), Samborondón 091650, Ecuador

**Keywords:** conservation, invasive alien species, management, Pyrenees

## Abstract

Biological invasions are among the most pervasive threats to biodiversity, ecosystem functioning, and human well-being. Despite international policy efforts, the number of introductions continues to rise worldwide. Mountains, once considered resistant to biological invasions due to harsh climates and isolation, are becoming increasingly vulnerable. Human activities—tourism, infrastructure development, and land-use change—combined with climate warming, are creating new pathways and suitable conditions for non-native plants to spread upslope. Global evidence shows a rapid increase in alien species richness in mountain ecosystems, with some taxa shifting elevation by hundreds of meters. The problem of biological invasions becomes critical when considering that mountains harbor nearly a quarter of the planet’s total biodiversity. This issue is even more concerning in biodiversity hotspots such as the Mediterranean Basin, where mountains present an exceptionally high rate of endemism and have served as glacial refugia. The Pyrenees exemplify this dynamic: historically shaped by millennia of human activity, they now face growing pressures from tourism and climate change. Recent cataloging efforts reveal 771 alien taxa, surpassing figures for larger ranges like the Alps. These findings challenge long-held assumptions about mountain resilience and underscore the urgent need for coordinated monitoring, early detection, and management strategies—including citizen science initiatives—to mitigate ecological impacts and protect mountain biodiversity under accelerating global change.

## 1. The Increasing Problem of Plant Invasions

Biological invasions are increasingly recognized as one of the most pervasive threats to natural systems, with significant consequences for biodiversity, human well-being, and economic stability. Evidence accumulated over the past decades shows that invasive alien species (IAS) are responsible, solely or along with other drivers, for a significant proportion of global extinctions—around 60%, according to recent assessments [1]. Beyond causing species extinctions, IAS can exert a wide range of impacts on native ecosystems, including losses of genetic diversity, reductions in population size and fitness, range shifts, and alterations at the community level (e.g., changes in species diversity and abundance) [2]. These impacts may result from one or multiple mechanisms, such as competition, allelopathy, hybridization, or modifications of the abiotic environment, including changes in soil pH, nutrient availability, light conditions, and disturbance regimes [3].

In addition to their direct impacts on native biodiversity, biological invasions impose substantial economic costs worldwide (collectively, the reported costs of invasions reached a minimum of USD 1.3 trillion over the period 1970–2017 [4]), with alien plant species contributing significantly through their impacts on agriculture, forestry, and ecosystem services. In agricultural systems, invasive plants reduce crop yields and increase management and control costs, resulting in considerable economic losses [5,6]. In forest ecosystems, alien plants can hinder forest regeneration, increase fire risk, and reduce timber productivity, thereby affecting both commercial forestry and associated livelihoods [7,8]. Beyond these impacts, alien plants also generate substantial indirect costs through the degradation of ecosystem services, including water regulation, soil stabilization, carbon sequestration, pollination, and recreational and cultural values [9,10]. Invasive alien plants can also have substantial impacts on human health, as many species are toxic or poisonous to humans, including allergenic plants, species that cause contact dermatitis, and plants armed with thorns, spines, or prickles [11]. For example, *Ambrosia*-induced allergies in Europe have been estimated to generate annual costs of approximately EUR 7.4 billion, when accounting for both medical treatment expenses and productivity losses due to lost working time [12].

Unfortunately, the arrival of new alien species shows no signs of slowing; on the contrary, introductions continue to rise worldwide [13], and projections suggest that more than 200 additional species may be added annually if current trends persist [1]. Specifically for plants, it has been predicted that the number of non-native plant species will increase by 6% to 41% from 2005 to 2050 depending on the continent (excluding the Pacific Islands) [14]. In angiosperms, the doubling time of the number of new records is estimated at just 17 years [15].

Human activities—whether deliberate, as in agriculture, forestry, or horticulture, or accidental, through contamination pathways—have facilitated the movement of plant species far beyond their native ranges [16,17]. Once established, these species interact with landscapes already undergoing rapid transformation. Habitat degradation, fragmentation, and land-use change are reshaping ecosystems across biomes, reducing their resistance to invasion and increasing their vulnerability [18,19]. Consequently, IAS have become prominent components of many floras, contributing to the reorganization of ecological communities and the erosion of native biodiversity. In some regions, their dominance is particularly striking; in New Zealand, for instance, the non-native naturalized species constitute nearly 44% of the country’s plant diversity [20].

The growing recognition of these impacts has prompted international policy responses. The Kunming–Montréal Global Biodiversity Framework (KMGBF) of the Convention on Biological Diversity (CBD) places IAS at the center of one of its main targets. Target 6 calls to “eliminate, minimize, reduce and/or mitigate the impacts of invasive alien species on biodiversity and ecosystem services by identifying and managing pathways of the introduction of alien species, preventing the introduction and establishment of priority invasive alien species […]” [21]. Achieving this target requires coordinated global action, robust biosecurity measures, and effective restoration strategies, alongside public awareness and stakeholder engagement. Comprehensive and up-to-date catalogues of IAS are essential tools for understanding the scope of invasions within a territory, guiding management priorities, and informing policy decisions. Systematic inventories of alien species provide the baseline information necessary for Early Detection and Rapid Response (EDRR) frameworks [22] by allowing newly introduced or expanding species to be identified at an early stage. Early detection significantly increases the likelihood that management actions will be effective and cost-efficient, as eradication or containment is more feasible before invasive populations become widespread. For example, EDRR have shown notable success in the control of *Opuntia aurantiaca* invasion foci in Spain, whose early detection—in some cases, by means of citizen science tools [23]—have prevented its establishment in its potential areas [24].

## 2. Mountains: Plant Heavens Threatened by Biological Invasions

Until recently, mountains were widely considered to be relatively immune to biological invasions. Harsh climatic conditions, such as low temperatures, short growing seasons, and strong environmental filtering along steep elevational gradients, were thought to limit the establishment and spread of alien species. In addition, the geographical isolation of many mountain areas and lower levels of human disturbance compared to lowlands (i.e., a much-decreased human-mediated propagule dispersal) reinforced the idea that high-elevation ecosystems were naturally protected from invasions [25,26,27]. This assumption was probably magnified by the overwhelming number of studies carried out in lowland areas compared to mountain ones [26], a situation that is slowly changing thanks to the launch of the Mountain Invasion Research Network (MIREN [28]) in 2005. This is a global effort to apply the principles of plant invasion ecology to mountainous environments and focuses primarily on monitoring alien plant invasions globally [29].

**Figure 1:**
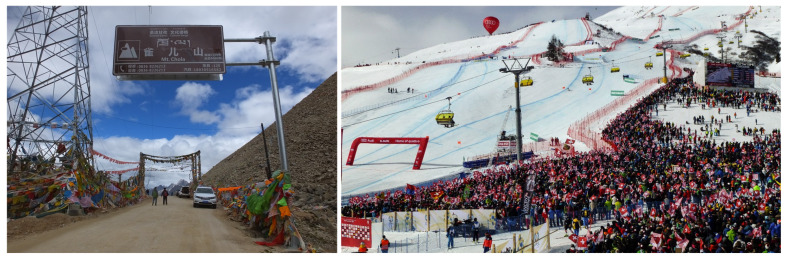
Anthropogenic development of mountain areas is increasing the risk of biological invasions. (**Left**), Chola mountain pass (W Sichuan, China), at 5050 m a.s.l., one of the highest in the region (photograph: J. López-Pujol). China planned to build or upgrade 100,000 km of rural roads (of which a large part is in mountainous areas) just during the year 2025 [52]. (**Right**), a ski resort in St. Moritz, Switzerland (photograph: Wikipedia [53]). Globally, there are over 5700 ski resorts [36].

However, the perception of mountains as environments “free of invaders” has changed markedly in recent years. The intensification of human activities in mountain regions—including tourism, transportation, and land-use change (e.g., overexploitation of natural resources; Figure 1)—has facilitated the introduction and spread of alien species (e.g., [30,31,32,33,34,35]). Mountain tourism is estimated to account for between 195 and 375 million international arrivals, based on 2019 figures [36]. Roads, railways, and trails fragment habitats and enhance resource availability by disturbing vegetation through construction, trampling, and grazing [37,38], thereby lowering biotic resistance and facilitating the establishment of non-native species. While there is increased propagule pressure near communication infrastructure and tourism/recreational areas [39,40,41,42,43,44], seeds can additionally be transported upslope by cars, hikers, livestock or wild animals [45,46].

At the same time, climate warming will reduce thermal constraints at high elevations, allowing alien species to survive, reproduce, and spread upslope. While most invaders are spreading rapidly to higher altitudes simply because they are filling their climatic niche, warming temperatures will allow alien plants to spread to even higher elevations in the future as their ecological niche is also shifting upwards [35]. This concern is amplified by growing evidence that rates of climate change (measured as changes in temperature, precipitation, and snowfall) are higher in mountain regions than in lowland areas [47]. Consistent with this pattern, species distribution models (SDMs) under climate warming scenarios indicate that rising temperatures could enable IAS to expand in altitude anywhere (e.g., [48,49,50,51]).

**Figure 2:**
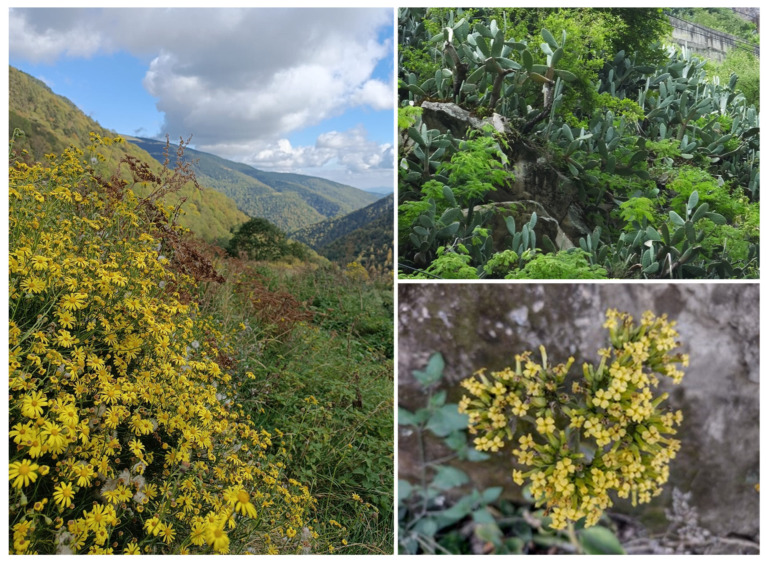
Some examples of alien species invading mountainous areas far from their native ranges. (**Left**), *Senecio inaequidens* (native area: S Africa), near Vilallonga de Ter (Girona, Spain), in the Pyrenees (1540 m a.s.l.) (photograph: E. López-Guillén). (**Upper right**), *Opuntia ficus-indica* (native area: N America), near Kangding City (Sichuan, China), Hengduan Mountains (1700 m a.s.l.) (photograph: J. López-Pujol). (**Lower right**), *Kalanchoe densiflora* (native area: C and E Africa), near Chimborazo, Ecuadorian Andes (3300 m a.s.l.) (photograph: J. López-Pujol).

As a result of the abovementioned factors, biological invasions in mountain ecosystems have been increasingly documented (Figure 2), particularly over the last 15–20 years. This trend challenges the long-held assumption that mountains are resistant to invasion and underscores the need for greater attention to invasion dynamics at high elevations. The seminal work of Pauchard et al. (2009) [26] was the first to explicitly question the notion that mountain ecosystems are not at risk from invasive plants, reporting the alarming finding that more than one thousand non-native plant species were already naturalized in high-elevation ecosystems worldwide at that time, based on unpublished data from MIREN. Since then, there has been an explosion of studies of plant invasions into mountain areas, with some trying to detect global-scale patterns as well as more local processes. For example, Seipel et al. (2012) [54], also based on MIREN data, showed that alien species richness is generally higher in New World regions and consistently declines with increasing distance from roadsides across regions. Similarly, McDougall et al. (2011) [55] identified clear patterns in the composition of non-native mountain floras, which are typically dominated by herbaceous species with an approximately equal representation of annuals and perennials. They also demonstrated that most alien plants in mountain regions originate from lowland environments and exhibit broad climatic tolerances, rather than being mountain (stress-tolerant) specialists in their native ranges. McDougall et al. (2018) [56] demonstrated that alien plants that spread along roadside dispersal corridors differ in species traits from those spreading into adjacent natural vegetation (being the first long-lived, non-ruderal species without seed dispersal traits, whereas the second are shade and moisture-tolerant). Even more striking are the findings of Iseli et al. (2023) [35], who reported a global average increase of 16% in the number of alien plant species across mountain regions over the past decade.

Local-scale studies have also provided noticeable results that corroborate the global trend of mountains increasingly exposed to biological invasions. In China, for example, the average maximum elevation of invasive Asteraceae (17 species in total) increased from 2150 m to 2420 m between 2016 and 2020 [57]. Such upward shifts will continue in the future as SDM-based studies reveal, although these elevation changes are regionally dependent; for instance, Petitpierre et al. (2016) [48] found a less pronounced shift in New South Wales (Australia) compared to Switzerland when predicted for two time periods (2030 and 2070). Local surveys of alien flora can also be highly effective for detecting ongoing invasions when conducted repeatedly over time. For instance, a recent study on Tungurahua Volcano in the Ecuadorian Andes recorded up to 38 alien taxa, whereas no alien species had been observed at the site 30 years earlier [58].

The substantial influx of IAS into mountain areas is even more concerning when considering the enormous biodiversity value of mountain ecosystems. Mountains occupy roughly 22–25% of the Earth’s land surface, yet they are recognized for their remarkably high levels of species richness and endemism [59,60]. They contribute disproportionately to global terrestrial biodiversity, hosting over 85% of the world’s amphibian, bird, and mammal species—a phenomenon referred to by some authors as “Humboldt’s enigma” [60]. Furthermore, the majority of the world’s 36 biodiversity hotspots [61] are located wholly or partly within mountainous regions. The extraordinary biodiversity found in mountain ecosystems results from the interplay between topography and climate. Sharp environmental gradients generate diverse microclimates that promote species turnover and specialization, while complex and rugged terrain enhances isolation and drives speciation [62,63,64].

This interplay between topography and climate has also allowed mountains to play a crucial role as refugia for biota during periods of global cooling throughout the Neogene and Quaternary [65,66,67]. Mountains served as important refuges for plant species primarily for two reasons: (i) they would have experienced relatively stable eco-climatic conditions during Quaternary climatic oscillations, largely due to sustained moisture availability and heterogeneous topography that provided sheltered environments [65,68,69]; and (ii) they would have enabled plants to respond to climatic shifts by moving along elevation gradients during warm interglacial and cold glacial periods, rather than undertaking extensive latitudinal migrations [70,71,72]. In recent years, there has been some empirical evidence of these mechanisms acting in several mountainous areas [73,74,75,76]. Because of this role as refugia, populations of native plants within mountains often show high levels of genetic diversity and significant amounts of unique haplotypes/alleles (e.g., [66,77]).

**Figure 3:**
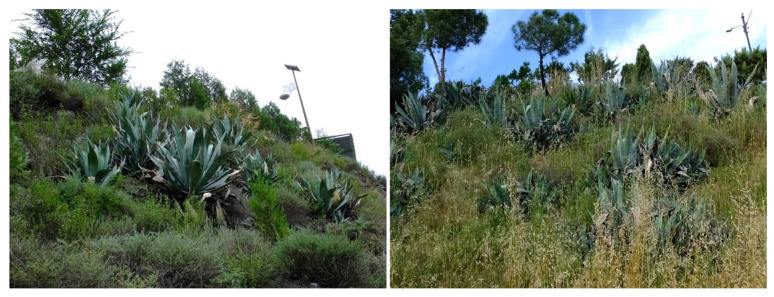
An example of landscape homogenization caused by invasive alien species. The presence of the magnificent *Agave americana* makes two landscapes superficially resemble each other when they should be very different. (**Left**), Guchengcun, Li County (Sichuan, China), Hengduan Mountains, 1500 m a.s.l. (photograph: J. López-Pujol). (**Right**), Biosca, Segarra County (Catalonia, Spain), Pyrenees, 450 m a.s.l. (photograph: J. López-Pujol). The Chinese site corresponds to cold-adapted mixed forests with conifers and broad-leaved trees, whereas the Spanish site represents much warmer sub-Mediterranean oak and pine forests.

In addition to their ecological value, mountain ecosystems provide key services for human well-being, including water supply and carbon sequestration [78,79,80], but also aesthetic, spiritual, and cultural ones [81,82,83]. Among the latter, landscape homogenization (Figure 3)—driven by biological invasions, which lead to the global homogenization of biological diversity [84]—could result in a form of “biocultural homogenization” [85].

## 3. Plant Invasions in Mediterranean Mountains: The Pyrenees as a Case Study

The Mediterranean Basin is widely recognized as a biodiversity hotspot, characterized by exceptionally high plant diversity and a considerable number of endemic species [86,87]. Within this region, mountain systems not only occupy a significant proportion of the territory in several Mediterranean countries but also harbor much of their biodiversity [88]. Notably, all of the local plant diversity hotspots within the Mediterranean basin (which were identified by F. Médail and P. Quézel almost 30 years ago) are essentially mountainous, with exceptional rates of endemism of up to 50% in some areas [89]. Furthermore, the majority of the 52 glacial refugia identified for the entire basin (based on genetic and phylogeographic data) by F. Médail and K. Diadema (2009) are located in mountainous regions [66].

However, unlike other regions where mountain ecosystems have remained relatively intact until recent times, Mediterranean mountains have been profoundly shaped by human activities over millennia [83,90]; see [91] for visualizing the anthropogenic ecological transformations over time of the varied mountainous areas in the world. These long-standing anthropogenic impacts—now intensifying—combined with climate change, which is expected to severely affect the Mediterranean Basin and its mountain environments [92], pose serious challenges for the conservation of these ecosystems. In turn, this makes the Mediterranean Basin particularly vulnerable to biological invasions [93]. The combination of high connectivity, intense human activity, and climatic conditions favorable to many alien species has already facilitated their introduction and spread into high-altitude Mediterranean environments. Invasive plants such as *Ailanthus altissima*, *Robinia pseudoacacia* and *Senecio inaequidens* (see a picture of the latter in Figure 2) are already altering native plant communities in several Mediterranean mountain systems [94,95]. For example, in Vesuvius Volcano National Park (southern Italy), *R. pseudoacacia* has spread widely after disturbances such as wildfires, colonizing *Castanea sativa* stands and potentially displacing native vegetation through vigorous sprouting and root suckering [96]. Although Mediterranean mountains are currently less affected by invasive species than lowlands, which host most invasion hotspots, this situation will change significantly in the near future. Multispecies SDMs indicate that invasions will become a serious issue in mountainous areas in the northern part of the Mediterranean Basin [97].

Situated at the interface between the Mediterranean and Eurosiberian regions, the Pyrenees host an exceptional floristic richness, with at least 3654 native taxa according to the *Atlas of the Flora of the Pyrenees* [98]. This mountain range represents a paradigmatic example of the current dynamics of plant invasions in Mediterranean mountains. As in other mountain systems of the Mediterranean Basin, the Pyrenees have been subject to anthropogenic impacts since ancient times (with human presence in valleys and lands of slightly over 1000 m a.s.l. at the end of the last glacial period and in alpine and subalpine areas after the Younger Dryas [99]). Traditionally, human activities in this area were linked to agriculture and livestock farming; however, in recent decades, the region has undergone a marked process of depopulation while its economy has shifted towards tourism. Consequently, Pyrenean ecosystems now face new pressures, including the expansion of road networks, overtourism and urban sprawl, as there is an accelerated construction of holiday chalets, especially in the eastern Pyrenees [100]. Like many other mountain systems in the region, the Pyrenees are expected to be strongly affected by climate change, with projected impacts such as rising mean temperatures [101], increasing aridity and desertification [102], and altitudinal shifts in vegetation [103]. These environmental changes, combined with ongoing human disturbances, are creating conditions that facilitate the introduction and establishment of non-native plant species.

Although the flora of the Pyrenees has been studied for centuries [104], an integrated catalogue of the entire mountain range was only recently completed, thanks to several projects funded by the European Regional Development Fund (ERDF) through the INTERREG POCTEFA program [105]. Initially, the *Atlas of the Flora of the Pyrenees* [98] reported 462 alien taxa, but subsequent revisions increased this number to 601 taxa [106]. As part of the current POCTEFA project FLORAPYR 3D (2024–2026), now at its midpoint, the list has been further updated through the incorporation of recent records from the French side and the correction of erroneous data. As of today, the catalogue of alien flora in the Pyrenees includes 771 taxa, a remarkable figure compared to other mountain ranges (e.g., the Alps, which host far fewer taxa despite their larger area [107]). This exceptional diversity can be attributed—at least in part—to the high variability of climatic, topographic, geological, and ecological conditions that characterize the region. Notably, a significant portion of the Pyrenees includes habitats with a pronounced coastal influence—indeed, some areas literally descend to the sea, as in Empordà at the eastern extremity—which allows floristic elements from mild or even relatively warm climates (e.g., Crassulaceae and Cactaceae) to thrive in many low-mountain areas. The human factor, however, plays a major role in the high number of alien species recorded in the Pyrenees. Certain parts of the Pyrenees, particularly in the east, coincide with areas of intense tourist activity (northern Costa Brava/Cap de Creus and surrounding regions), while others are located very close (<50 km) to one of southern Europe’s largest metropolitan regions, Barcelona, with over 5.5 million inhabitants (see [106] for more details).

**Figure 4:**
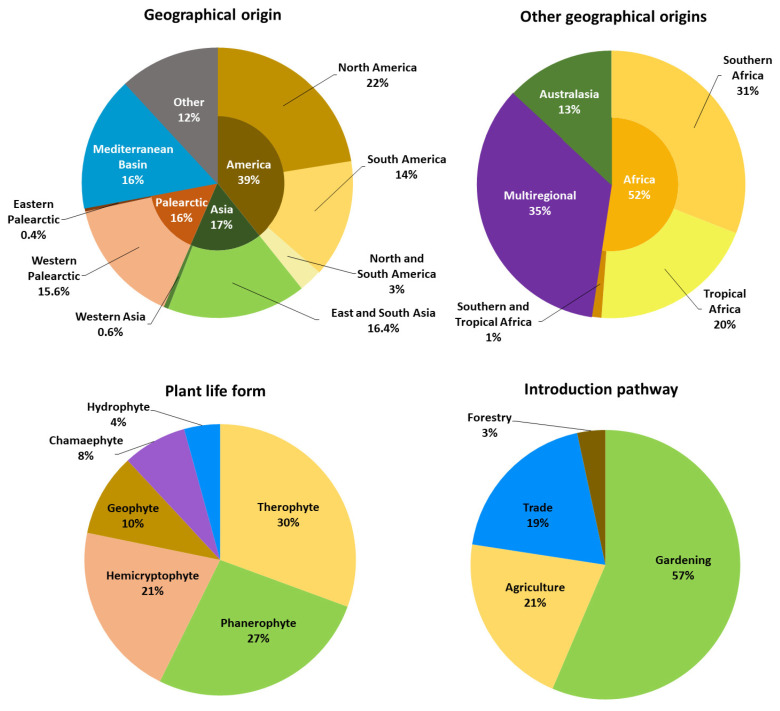
Graphics representing geographical origin, introduction pathway and plant life forms of alien plant taxa of the Pyrenees. In relation to geographical origin, taxa with no clear geographical origin (hybrid and cultivated taxa, 53 in total) were excluded. The classification of geographic origins and introduction pathways is based on Aymerich and Sáez (2019) [109].

Although some species will still be incorporated into the final catalogue (see below), a preliminary analysis of the available data allows us to tentatively conclude that the alien flora of the Pyrenees is characterized by three features. First, there is a predominance of species of American origin, which account for more than one-third of the total, with North America contributing more taxa than South America, followed by East and South Asia, the Mediterranean Basin, and Western Palearctic (Figure 4). That the Americas constitute the main region of origin of the alien flora of the Pyrenees is not surprising, as this region is also the principal donor of alien plant species to both Spain [108] and Catalonia [109], territories that encompass large portions of the Pyrenean range. However, in contrast to these areas, the alien flora of the Pyrenees includes a higher number of North American than South American species, likely because many North American plants are pre-adapted to the colder climatic conditions of the Pyrenees.

Second, there is a dominance of therophytes as the main plant life form (ca. 30%), followed by phanerophytes (ca. 27%) and hemicryptophytes (ca. 21%) (Figure 4). The clear dominance of herbaceous species is a common pattern in mountain alien floras worldwide, with a mean proportion exceeding 90% [55]. The relatively high proportion of woody species in the Pyrenean alien flora (over one quarter) warrants further investigation, but it may be partly explained by the strong Mediterranean influence affecting large portions of these mountains, particularly its eastern sector. Mediterranean-type climate can impose strong environmental filters (summer drought, high climatic variability) for which woody plants often possess traits such as longevity, resprouting ability, and stress tolerance that increase their chances of establishment under these conditions. Consistently, phanerophytes are well represented among alien species in Mediterranean Europe (almost one quarter [110]), and have also been reported at high proportions in the alien floras of several Mediterranean mountain areas, such as Calabria (ca. 30% [111]).

Third and last, there is a prominence of gardening as the principal pathway for the introduction of alien plants into the Pyrenees (ca. 56%), followed by agriculture (21%), trade (19%), and, to a much lesser extent, forestry (ca. 3%) (Figure 4). The clear dominance of ornamental uses among plant introduction pathways is consistent with global patterns observed in mountainous regions, where 57% of species with documented uses are ornamentals [55], as well as with global invasion patterns more broadly [112].

For the second part of the FLORAPYR 3D project, the incorporation of all citizen science data being collected through the specially created iNaturalist project [113]—enhanced by several activities organized by our team to date, including several bioblitzes—is still pending. Although the overall increase in the total number of species, thanks to citizen-contributed data, may not be very large, we do expect a considerable increase in the number of records for individual taxa. As an example, a simple comparison between the *Atlas* records and those from iNaturalist for a well-known species such as *Senecio inaequidens* indicates that data collected through the citizen science portal could increase the number of UTM (Universal Transverse Mercator) grid cells with the presence of this South African invasive species by nearly one-fifth.

The categorization of all recorded taxa according to their invasion stage (that is, determining whether a given taxon has become naturalized and, if so, whether it exhibits invasive behavior) is also still pending. This process will facilitate the identification of dispersal and establishment patterns for each taxon, which in turn will contribute to a better understanding of the ecological risks associated with their presence. Such classification will also be key for prioritizing management actions, thereby improving the design of detection, control, and eradication strategies. In sum, this catalogue provides the first detailed and comprehensive overview of biological invasions in the Pyrenees and, even though it is not yet complete, it already challenges the long-standing assumption that mountain habitats are relatively resistant to biological invasions.

## 4. Recommendations

Despite recent progress in the study of mountain alien flora, many gaps remain at both regional and impact scales. While research on mountain ecosystems worldwide is justified by the global increase in the establishment of alien taxa in mountainous areas [35], several geographic regions still lack studies or have clearly insufficient data, such as much of Africa, Central Asia, and Central and South America. Although the Andes represent the world’s longest mountain chain (>7000 km) and harbor the highest number of plant species (approximately 30,000 [114]), they have received relatively little attention. Evidence of this is provided by a recent compilation of the alien flora of the Andes based on published literature up to mid-2023, which reported only 219 plant species [115]—a surprisingly low figure. Furthermore, the recent publication of the catalogue of alien flora of continental Ecuador reported 451 plant species just for the Ecuadorian Andes [116], highlighting that the Andean non-native flora remains clearly understudied.

Regarding the ecological impacts of invasive plant species, there are large biases for certain types of habitats. According to Vilà et al. (2024) [117], the least represented habitats in impact studies are deserts and xeric shrublands, high mountains, and subtropical forests. In the context of climate change, it is imperative to focus more on the impacts of invasive plants in mountain regions, as warming is expected to enhance their dispersal and establishment at higher altitudes, making this an area of critical concern [118]. Emerging frameworks for assessing the negative impacts of alien species on native biodiversity, such as EICAT, may help to address this gap. EICAT, which stands for Environmental Impact Classification for Alien Taxa, is a global standard recently proposed by the IUCN. Conceptually and structurally modeled on the IUCN Red List of Threatened Species, it aims to categorize alien species according to the magnitude of their impacts—from minimal to massive—based on documented evidence of environmental harm [3].

While EICAT can facilitate communication between research teams and also with stakeholders and decision-makers, cooperation tools are needed when targeted mountain systems are split into different territories, as often occurs. As varied regions usually face similar problems regarding IAS, raising awareness and cooperating on an international scale has become crucial to manage and prevent biological invasions, especially those with a massive impact on high-elevation ecosystems. In this sense, MIREN has been an absolute success in implementing a shared methodology designed to monitor plant invasions and climate change impacts in mountains globally; at present, over 20 mountain regions from all continents (except Antarctica) have been surveyed and cover the major climatic zones and include island and continental systems. The INTERREG POCTEFA program, within the framework of which the catalog of alien flora of the Pyrenees is being developed, is an example of good performance for a single mountain range shared between different political entities (Andorra, France, and Spain).

Early detection and a deeper understanding of the distribution could also be helpful to control the expansion of invasive alien taxa across and between mountain systems. To do so, iNaturalist is one of the most used tools, presenting a cost-effective way to further enhance alien plant records and outreach campaigns that highlight mountain areas [119]. Opportunistic iNaturalist observations have the potential to complement and expand professional invasive plant monitoring, which was often affected by inverse sampling biases. Invasive species represent a high proportion of iNaturalist plant observations and are recorded in environments that sometimes were not captured by professional surveys [120], therefore expanding the knowledge of biological invasions in mountain areas.

## Data Availability

The original contributions presented in this study are included in the article. Further inquiries can be directed to the corresponding author.
